# Experiences with alternative online lectures in medical education in obstetrics and gynecology during the COVID-19 pandemic—possible efficient and student-orientated models for the future?

**DOI:** 10.1007/s00404-021-06356-5

**Published:** 2021-12-28

**Authors:** Maximilian Riedel, Gabriel Eisenkolb, Niklas Amann, Anne Karge, Bastian Meyer, Maria Tensil, Florian Recker, Anna Maria Dobberkau, Fabian Riedel, Bettina Kuschel, Evelyn Klein

**Affiliations:** 1 Department of Gynecology and Obstetrics, Klinikum rechts der Isar, Technical University, Munich, Germany; 2 Department of Gynecology and Obstetrics, Ludwig Maximilians University (LMU), Munich, Germany; 3 Kirinus Clinic Schwabing, Munich, Germany; 4 Department of Gynecology and Obstetrics, Bonn University Hospital, Bonn, Germany; 5 Department of Gynecology and Obstetrics, Heidelberg University Hospital, Heidelberg, Germany

**Keywords:** COVID-19, Medical education, e-learning, Learning behavior, Lectures, Remote learning

## Abstract

**Purpose:**

The onset of the COVID-19 pandemic posed an eminent challenge for medical teachers worldwide. Face-to-face lectures and seminars were no longer possible, and alternatives had to be found. E-learning concepts quickly emerged as the only practicable solutions and also offered the opportunity to evaluate whether traditional face-to-face lectures could be translated into an online format, independent of the COVID-19 pandemic.

**Methods:**

We offered an e-learning program consisting of lecture notes, screencasts with audio narration, and online webinars that covered topics normally taught in traditional lectures and seminars. To evaluate the learning behavior and quality of our e-learning program, we drafted a questionnaire that students completed at the end of the 2020 summer semester that had been designed to enable a comparative analysis of the different e-learning modules.

**Results:**

Voluntary participation in the online courses was high. Survey analysis revealed high satisfaction with and a distinctive preference for the format, even under regular, COVID-19-independent conditions. In general, a positive appraisal of e-learning—especially as a substitute for regular lectures—was found. Students also reported higher studying efficiency. Exam results were equal to those of previous semesters.

**Conclusion:**

Both acceptance of and satisfaction with our e-learning modules were high, and students displayed increased demand for this kind of e-learning format. We, therefore, conclude that e-learning offerings could serve as reasonable, efficient, student-orientated substitutes for certain medical courses, especially lectures. These curricular adaptations would correlate with the high digitalization seen in students’ everyday lives. This correlation may also hold true independent of the ongoing COVID-19 pandemic.

## Introduction

Before the COVID-19 pandemic, digitalization in medical studies had only slowly gained relevance in Germany. Digital media and e-learning concepts had functioned as an addendum rather than an integral part of the regular curriculum, especially with regard to the organization of teaching and the provision of complementary teaching resources [1, 2]. Most medical teaching at German medical faculties is still executed in a manner comparable to that of the pre-digital era, with scheduled face-to-face lectures and seminars. By contrast, students’ preference for online learning over learning via analogous media has increasingly accelerated in recent years [3, 4]. Commercially available online medical learning platforms, such as AMBOSS [5], Thieme Examen [6], and Medi Learn [7], have increased in popularity among German medical students and physicians. These platforms provide comprehensive and interlinked online knowledge databases as well as multiple-choice questions that prepare students for the nationwide German Medical State Examination [8].

Face-to-face lecture-style teaching is an efficient and well-established method of disseminating core information to a large audience [9]. Nonetheless, efforts have been made internationally in the last decade to transform medical teaching by reducing the number of lectures and simultaneously implementing more self-directed learning that promotes individualized education or uses (online) technology to improve education [10, 11]. A similar concept has been pursued in Germany via the reform of medical studies in line with the “2020 master-plan medical studies” [12]. One of the plan’s major aims is to promote more practical, patient-orientated, and integrative teaching that is compensated by a reduction in classical lectures [13]. The benefits of small-group teaching that focuses on competency-based education and self-directed learning skills are eminent [14]; however, they are also highly time-consuming for the students who have had to cope with the intense growth of the medical curriculum in the last decade [15]. It is, therefore, unclear whether traditional in-person lectures on fixed dates during the semester are still time-efficient, student-orientated, and appropriate enough for modern medical education.

The COVID-19 pandemic has posed new and unprecedented challenges for medical students and medical education in general [16]. The complete disruption of medical education has served as a catalyst for the advancement of online learning tools both in Germany [17, 18] and worldwide [19–23]. Students’ generally positive reception of e-learning thus begs the question as to which areas of medical teaching not only enable a digital transformation of the face-to-face curriculum but are also overdue for such a change and thereby render it reasonable to maintain, even after the pandemic [24].

The COVID-19 pandemic has provided an opportunity to test different online teaching resources as a substitute for lectures. Our course offered online alternatives to traditional face-to-face lectures and seminars in the format of lecture notes, screencasts with audio narration, or online live webinars. The extent to which this massive boost of e-learning during the pandemic should continue and influence future medical education remains to be decided. We hypothesized that medical students value e-learning favorably and would endorse its broader implementation in the standard curriculum, because it reflects the students’ natural use of media in their everyday lives [25]. Therefore, we aimed to address three different questions in our survey: (I) What are our students’ preferred learning environments and their typical studying behavior? (II) How satisfied are they with adopting our online resources as a substitute for lectures? (III) Can e-learning contribute to a more-time-efficient learning environment after the restrictions imposed by the COVID-19 pandemic have been lifted?

### Project description

The COVID-19 pandemic offered the opportunity to test and evaluate new online teaching concepts within the compulsory curricular course of obstetrics and gynecology. Students enrolled in their 8th semester of medical studies in the 2020 summer semester at the medical faculty of the Technical University Munich participated in the course. Clinics were given autonomy to conceptualize and implement the teaching concepts to account for the individual character of each subject.

The main goal of our study was to evaluate the extent to which a concise transfer of knowledge from a clinical expert to students—as is traditionally carried out via lectures—could be substituted by potentially more-time-efficient and student-orientated online-learning resources. Our learning modules did not cover other collaborative or more-interactive teaching resources, because their main intent was to focus on the transfer of knowledge in a passive lecture-style teaching format. We offered three e-learning modules that covered the topics of the course in obstetrics and gynecology that is normally taught in a series of face-to-face lectures (for approx. 150 students) and seminars (for 20–30 students). Bedside teaching and practical-skills training were not part of this year’s curriculum as they usually take place one semester later. All topics of the lectures and seminars were randomly transformed into lecture notes, screencasts with audio narration, or online webinars. The online live webinars were available on a given schedule, but all other learning resources could be downloaded from the central university education platform and worked on individually throughout the entire semester. No special order for completing the modules was stipulated. The three modules differed with regard to the extent of verbal illustration and narration, the possibility of interacting with the lecturer, and the opportunity for open discussion:

I. *Lecture notes*

The lecture notes took the form of PowerPoint slides, which are usually shown during presentations in face-to-face lectures and frequently downloaded for exam preparation, regardless of whether the students have attended the lecture. PowerPoint is a presentation program that is universally applied in medical studies for lectures and large-group teaching [26]. This module covered five comprehensive topics (family-related gynecological cancer; breast cancer screening and diagnosis; and breast cancer therapy, prognosis, and aftercare), each of which was equivalent to a 90-min lecture without further audio narration or any interaction with the audience or lecturer.

II. *Screencasts with audio narration*

Screencasts are digital recordings of computer-screen output. In our case, they included PowerPoint presentations with simultaneous audio narration from experts in the field that covered eight comprehensive topics (introduction; benign gynecological neoplasms; malign gynecological neoplasms of the uterus and cervix; the descent of pelvic organs; malign neoplasms of the ovary; gynecological emergencies; intrauterine growth restriction; and infections in gynecology and obstetrics), each of which lasted ca. 45–90 min. Screencasts have been found in the literature to be effective online-teaching instruments in operative dentistry studies [27], anatomy education [28], and preclinical medical studies [29]. This module was intended to simulate the common experience of a lecture with an oral presentation but without the need for or the intention for interaction with the lecturer, for example, by asking questions.

III. *Online live webinars*

We used the software ZOOM (by ZOOM Inc., 2020) for our webinars*.* Online meetings were planned as classical 90-min lectures at a fixed date and following a specific timetable during the semester and were delivered online for remote learning. Interactions with the audience and the lecturer were possible via chat messages and open questions. All meetings except one were recorded and could be downloaded without restriction. A record of attendance was not conducted. The following topics were covered: pregnancy and its physiologically adaptive changes; fetal surveillance and the detection of fetal distress; hypertensive disease during pregnancy, HELLP, pre-eclampsia; and regular birth- and intrapartum complications.

### Data acquisition

The 61-item questionnaire was disseminated following the multiple-choice exam at the end of the obstetrics and gynecology course in the 2020 summer semester. Study participation was voluntary and independent of the exam. All students provided written consent for participation. The questionnaire included 31 items with 5-point Likert-scale ratings that the participants used to indicate their agreement or disagreement with the statement for each item (1 = *strongly disagree (–)*, 2 = *disagree (-)*, 3 = *neither agree nor disagree (-/* +*)*, 4 = *agree (* +*)*, 5 = *strongly agree (*+ +*)*). Other questions were either dichotomous or classification questions. Results of the multiple-choice examinations from the obstetrics and gynecology course from the past 5 years were anonymously analyzed and compared with the current results. Examination questions were newly issued for each semester.

### Statistical analysis

The assessment was conducted via SPSS (IBM®). Tables and figures were generated in Word (Microsoft®) and GraphPad Prism (GraphPad® Software). *P* values were calculated using unpaired *t* tests. *P* values < 0.05 were defined as statistically significant.

## Results

### Characteristics of course participants and course evaluation

In total, 121 students (out of 150 total students in the semester; response rate: 81%) participated in the study. Our cohort represents the typical distribution of medical students in Germany in terms of sex (male: 31.4%; female: 68.6%) and age (86.8% between 20 and 25 years) [30]. More than two-thirds of participants lived in close proximity of (< 5 km) and needed less than 30 min to get to the university. Most students (67.8%) had no practical experience in obstetrics and gynecology beyond the compulsory courses. About half of participants were interested in obstetrics and gynecology; however, only 15% were considering obstetrics and gynecology for their later specialty training after graduation (Table 1).

**Table 1:** General characteristics of course participants and course evaluation

Item	Response
Age (*n* = 121)	< 20 years: 0%
	20–25 years: 86.8%
	26–30 years: 9.1%
	31–35 years: 1.7%
	> 35 years: 2.5%
Experience in obstetrics and gynecology beyond the standard curriculum (*n* = 121, multiple answers)	None: 67.8%
	Nursing placement: 16.5%
	Internship (Famulatur): 9.9%
	Elective (PJ): 0.8%
	Other: 5.5%
Distance between home and university (*n* = 121, shortest distance)	< 1 km: 4.1%
	1–2 km: 14.9%
	2–5 km: 49.6%
	5–15 km: 24.0%
	> 15 km: 7.4%
Time between home and university (*n* = 121, shortest distance)	< 5 min: 6.6%
	5–15 min: 34.7%
	15–30 min: 43.8%
	30–60 min: 13.2%
	60 min: 1.7%
Children living in the same household (*n* = 121)	Yes: 2.5% No: 97.5%
Interest in the specialty of obstetrics and gynecology (*n* = 121)	Very high: 11.6%
	High: 37.2%
	Average: 38.0%
	Low: 12.4%
	Very low: 0.8%
Chance for specialty training in obstetrics and gynecology (*n* = 121)	Very high: 2.5%
	High: 12.4%
	Average: 36.4%
	Low: 30.6%
	Very low: 18.2%
Main motivation for studying (*n* = 121, multiple answers)	Passing the exam: 22.3%
	Good grade: 48.8%
	Growth in knowledge: 61.2%
	Preparation for the state exam: 42.1%
	Other: 2.1%

Item	Mean Likert scale (1 = *completely disagree* – 5 = *completely agree*)
The course was well organized	3.9
The course was well structured	3.8
The modules progressed logically and expanded on previous material	3.8
I understood everything during the course	3.9
I learned a lot	3.7
The course covered all clinically relevant aspects of the specialty	3.7
The course covered all relevant aspects for passing the exam	3.6

Students’ satisfaction with the provided e-learning courses in terms of organization, technical application, relevance, learning success, and didactics was high, with median Likert scales for each item ranging from 3.6 to 3.9 (Table 1).

### Studying behavior before the COVID-19 pandemic

Half of the students attended less than 50% of non-compulsory lectures prior to the COVID-19 pandemic. Students reported diverse reasons for attending lectures and often indicated that receiving condensed and relevant content from a clinical expert as well as having personal interactions with fellow students were relevant factors. A minority (21.5%) considered posing questions to the lecturer to be important. Aside from lectures and seminars, students particularly preferred online self-study (e.g., AMBOSS) (71.9%) over analogous textbooks (38.8%). Condensed studying before the exam was favored over continuous studying throughout the semester (64.5%. vs. 34.6%). Forty percent of the students considered the optimal time of the day for learning to be the afternoon or night (Table 2).

**Table 2:** Learning behavior *before* the COVID-19 pandemic

Item	Response
How much of the non-compulsory courses do you usually attend during the semester? (*n* = 119)	0%: 3.4%
	0–25%: 30.3%
	25–50%: 25.2%
	50–75%: 21.8%
	75–100%: 19.3%
What are your preferred media or courses for studying? (*n* = 121, multiple answers)	Lectures: 66.9%
	Seminars: 68.6%
	Self-study, e.g., textbook, etc.: 38.8%
	Self-study online, e.g., AMBOSS®: 71.9%
	Study groups: 14.9%
	Podcast: 11.6%
	Online videos, e.g., YouTube®: 24.8%
How do you usually study during the semester? (*n* = 110)	Continuous studying throughout the semester: 34.5%
	Studying concentrated before the exam: 64.6%
	None/other: 0.9%
What type of learner do you consider yourself to be? (*n* = 121, multiple answers)	Visual (notes, mind maps, colored markers): 79.3%
	Auditory (podcast, reading loudly, lectures): 29.8%
	Communicative (study groups, discussions): 31.4%
	Haptic (own observation/experience, testing): 43.0%
	None/other: 5.0%
What is your preferred time of day for studying? (*n* = 121, multiple answers)	08:00–12:00: 63.6%
	12:00–16:00: 28.9%
	16:00–20:00: 19.0%
	20:00–24:00: 19.0%
	24.00–08:00: 2.5%
What is your main motivation for attending (non-compulsory) lectures? (*n* = 121, multiple answers)	Learning content from a clinical expert: 72.7%
	Learning the current state of research: 24.8%
	Posing questions to the lecturer: 21.5%
	Learning from clinical cases: 64.5%
	Learning content relevant for the exam: 71.2%
	Contact with fellow students: 75.2%
	Less time necessary for self-study: 27.3%

### Learning behavior during the COVID-19 pandemic

Half of the students completed more than 50% of their overall studying using solely the provided e-learning modules. The screencasts with audio narration and the live and recorded meetings were frequently and repeatedly used. In contrast, the lecture notes alone were less favored as only a minority (19%) of students used more than half of them. Students especially liked the flexibility of the e-learning modules; however, they also missed being present at the university and having direct interaction with fellow students or the lecturer. Again, posing questions was not relevant from the students’ point of view (Table 3).

**Table 3:** Learning behavior *during the* COVID-19 pandemic

Item	Likert scale (1 = *completely disagree* – 5 = *completely agree*) with mean
I usually ask questions during seminars (*n* = 121)	1: 35.5%
	2: 14.9%
	3: 12.4%
	4: 18.2%
	5: 19.2%
	Mean: 2.7
I usually ask questions during lectures (*n* = 121)	1: 47.1%
	2: 26.5%
	3: 9.1%
	4: 9.1%
	5: 8.3%
	Mean: 2.0
Lectures help me to structure my studying (*n* = 121)	1: 8.3%
	2: 10.7%
	3: 19.0%
	4: 28.9%
	5: 33.1%
	Mean: 3.7
I try to attend lectures as much as possible (*n* = 121)	1: 16.5%
	2: 12.4%
	3: 22.3%
	4: 18.2%
	5: 30.6%
	Mean: 3.3
Compulsory attendance of classes may contribute to better learning (*n* = 120)	1: 38.8%
	2: 24.8%
	3: 14.1%
	4: 12.4%
	5: 9.1%
	Mean: 2.4
Students should have learning autonomy as much as possible (*n* = 121)	1: 6.6%
	2: 7.4%
	3: 22.3%
	4: 24.8%
	5: 38.8%
	Mean: 3.8
Self-study can equivalently replace lectures and seminars (*n* = 121)	1: 33.1%
	2: 32.2%
	3: 25.6%
	4: 4.1%
	5: 5.0%
	Mean: 2.2

Item	Response
How much of the screencasts with audio narration did you work on? (*n* = 121)	0%: 8.3%
	0–25%: 8.3%
	25–50%: 10.7%
	50–75%: 14.9%
	75–100%: 57.9%
How frequently did you work on the screencasts with audio narration? (*n* = 119, multiple answers)	0 times: 14.3%
	1 times: 68.1%
	2 times: 14.3%
	> 2 times: 3.4%
When during the semester did you work on the screencasts with audio narration? (*n* = 110)	At the beginning of the semester: 9.7%
	In the middle of the semester: 24.8%
	At the end of the semester: 26.5%
	Continuously throughout the semester: 37.2%
	Other: 1.8%
What time of the day did you work on the screencasts with audio narration? (*n* = 121, multiple answers)	08:00–12:00: 53.7%
	12:00–16:00: 50.4%
	16:00–20:00: 33.1%
	20:00–24:00: 30.6%
	24.00–08:00: 2.5%
How much of the online ZOOM meetings did you attend live? (*n* = 121)	0%: 12.4%
	0–25%: 21.5%
	25–50%: 18.2%
	50–75%: 19.0%
	75–100%: 28.9%
How often did you work on the recorded ZOOM meetings (if available)? (*n* = 121)	0 times: 29.8%
	1 times: 57.0%
	2 times: 10.7%
	> 2 times: 2.5%
How often did you work on the PowerPoint slides/lecture notes? (*n* = 121)	0%: 10.7%
	0–25%: 55.4%
	25–50%: 15.7%
	50–75%: 17.4%
	75–100%: 0.8%
How much of your overall studying was done with the provided e-learning modules? (*n* = 119)	0%: 2.5%
	0–25%: 20.2%
	25–50%: 21.8%
	50–75%: 37.0%
	75–100%: 18.5%
Which media for studying did you use aside from the ones provided by the faculty? (*n* = 121, multiple answers)	Textbooks: 21.5%
	Online databases, e.g., AMBOSS®: 86.8%
	Online media (e.g., YouTube, podcasts): 22.3%
	Former exam questions: 84.3%
	Course script: 41.3%
	Study groups: 9.1%
Where did you work on most of the e-learning modules? (*n* = 119)	At home: 97.5%
	Library: 2.5%
How did you work on most of the e-learning modules? (*n* = 118)	Alone: 93.2%
	In pairs: 6.8%
	In a study group (*n* > 2): 0%
What did you like the most about the e-learning modules? (*n* = 121, multiple answers)	Flexible time expenditure: 93.4%
	Flexible learning environment: 70.2%
	Flexible repetition: 71.9%
	Flexible exam preparation: 59.5%
	Condensed learning: 55.4%
	Skipping non-relevant course topics: 65.3%
What did you miss the most about the e-learning modules? (*n* = 121, multiple answers)	Presence at the university: 55.4%
	Interaction with fellow students: 78.5%
	Direct person-to-person contact: 74.4%
	Asking questions: 28.1%
	Listening to questions: 38.0%
	Direct interaction with the lecturer: 60.3%

### Differential evaluation of e-learning courses

**Fig. 1:**
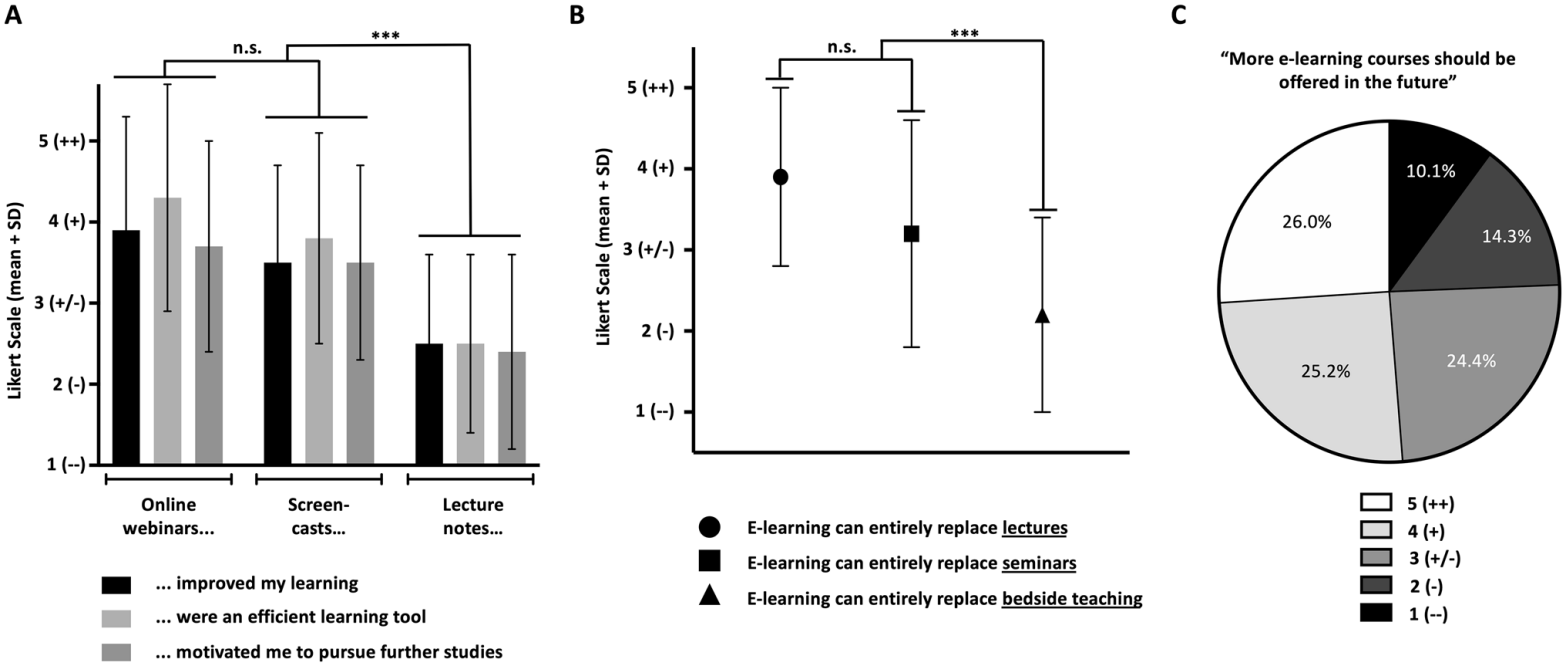
A, B Mean and standard deviation of Likert scale rating for the corresponding statement. (1 = “strongly disagree (--)”, 2 = “disagree (-)”, 3 = “neither agree nor disagree (+/−)”, 4 = “agree” ( +), 5 = “strongly agree” (++. *n.s*. = not significant, *** = *p* < 0,005 C Pie chart depicting the relative approval (in %) to the above statement

We found significantly better evaluations (*p* value < 0.001) of the online meetings and screencasts compared with the lecture notes in terms of improved studying, productivity, and motivation for further studies (Fig. 1A). From the students’ point of view, e-learning offerings were able to replace lectures to a high degree (Likert scale: 3.9; *p* value: < 0.001). By contrast, seminars (Likert scale: 3.1) and especially bedside teaching (Likert scale: 2.2) were considered less appropriate for e-learning (Fig. 1B). Accordingly, 51% of the students agreed with the statement that “more e-learning courses should be offered in future” (Fig. 1C).

### Course assessment and comparison of exam results

**Fig. 2:**
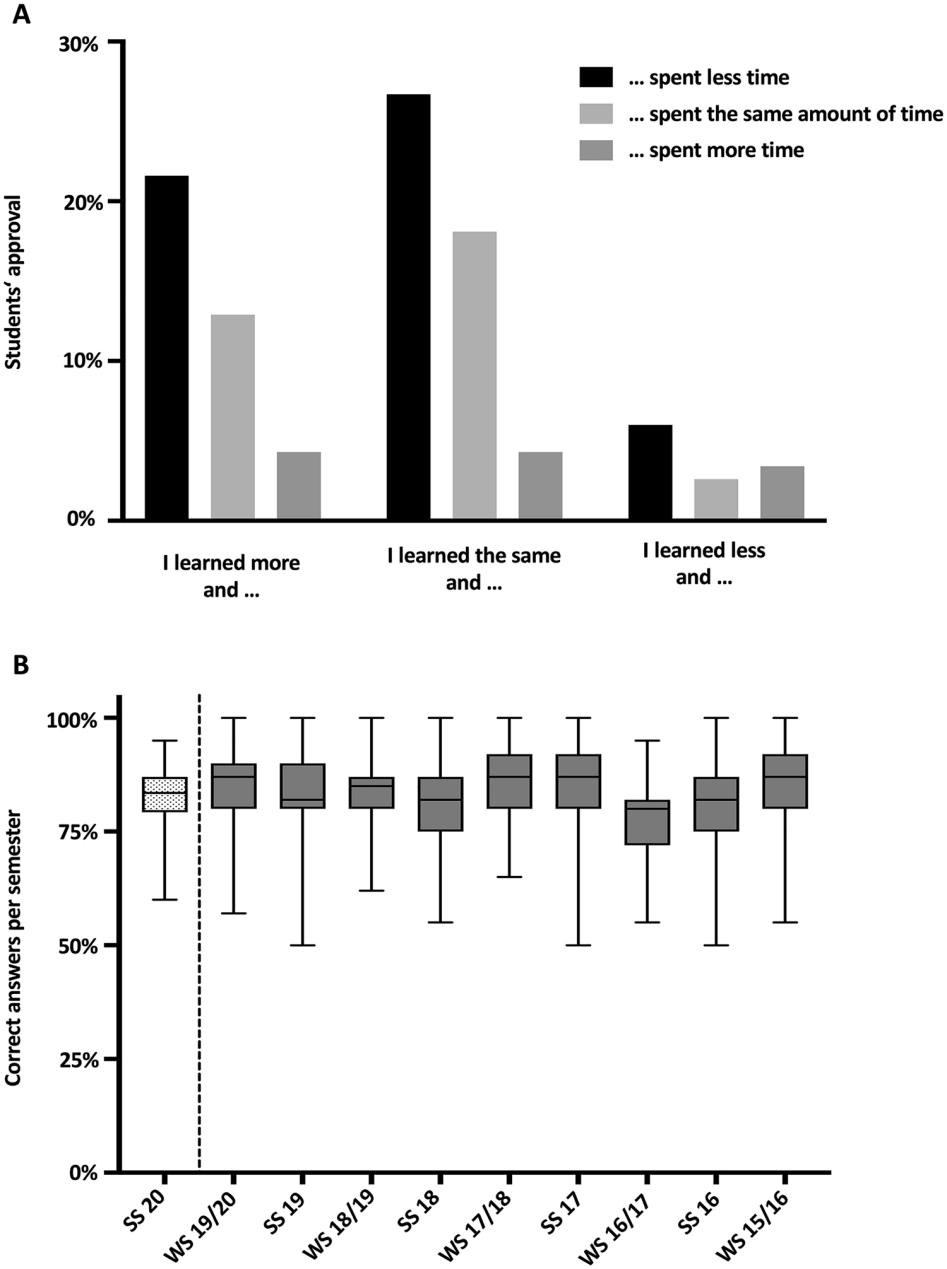
A Bar chart showing the relative approval (in %) of the statements relating the learning success to the time spent for learning. B Box-plot and whisker-plot showing the percentage of right answers of the current and past exams per semester (*SS *summer semester, *WS *winter semester)

More than 60% of students reported beneficial effects from our e-learning course: Learning improved in either less or the same amount of time, or an equal quality of learning was achieved in less time. Only 10% of students indicated that they had learned less, and 12% reported having a higher expenditure of time compared with a regular curriculum (Fig. 2A). We compared the current results of the final exam from the gynecology and obstetrics course during the COVID-19 pandemic with results of the previous 5 years and found the mean rate of correct answers during the year of study (83%) to be equal to the figure for previous years (83%) (Fig. 2B).

## Discussion

Our survey revealed that the current organization of face-to-face lectures does not align with the preferences of today’s generation of medical students for various reasons. Our students desire flexibility over being physically present for learning, as seen in the high appreciation of our online learning modules and the low participation rate of face-to-face lectures prior the COVID-19 pandemic. The fact that most lectures take place early in the morning and that they require continuous presence during the semester does not align with the preferences of a large proportion of our students. Bati et. al investigated reasons for non-attendance of lectures among Turkish medical-science students and found that sleeplessness and teaching inefficiency of lectures stood out [31]. As a learning method, however, lecture-style teaching was highly valued by our students. We conclude that the reported and frequently noticed low rate of attendance at lectures may not be due to the format itself, but rather to other external factors, such as the lack of flexibility of the lectures in terms of their scheduling and mode of delivery.

A major finding of our analysis was that direct interaction with the lecturer or the ability to ask questions were only relevant for a minority our students. By contrast, socializing with fellow students and friends was evaluated as being equally important during lectures. Likewise, Shah et al. reported that among American osteopathic medical students, a large proportion of time spent during lectures was used to study for other classes or was spent on social media or reading emails [32]. The benefits of compulsory attendance with or without being physically present in a lecture hall are, therefore, questionable. Our students differentiated between the requirements of small-group learning in seminars that relied on direct interaction among the attendees and the passive delivery of information in lectures. There was no significant difference between students’ appraisal of screencasts and online webinars. Therefore, lectures could be reasonably organized in a recorded (asynchronous) manner without weaking the relevant learning effect for the students. This finding could help to save faculty resources as recordings can be re-used for consecutive semesters, thereby leaving more time for practical and bedside teaching. Moreover, the assumed negative effects of online lectures—for example, distractions and less-active engagement in the lecture [33]—are not specific and also applied in our survey to face-to-face lectures. Most of our students lived close to their university (60% < 5 km). Saving time via remote learning was, therefore, only important for a small—albeit relevant—fraction of students due, for example, to childcare requirements, physical disabilities, or cost-efficient housing outside of expensive urban living spaces.

The advancement of e-learning has been remarkable in the last decade as it now constitutes part of the common learning activities of the current generation of students. E-learning technologies—such as screencasts and webinars—can be rapidly adopted and are associated with increased student and faculty satisfaction [34]. A majority of students use these technologies frequently as their major source of information in the clinic and for studying for multiple-choice exams [35]. Most of our students used AMBOSS or similar providers for their studies, while “analogous” media, such as textbooks, were only used by a fraction of students. In general, students in this semester appreciated the independent and flexible learning options, which generally support their need for autonomy and may lead to higher self-motivation and self-esteem [36]. Nonetheless, flexibility may also require a higher degree of learning discipline. A curriculum with more e-learning offerings and without a strict timetable could be more prone to neglect students with weaker learning skills and self-motivation. In that regard, the concept of self-regulated learning (SRL) has been established by educators and psychologists in recent years. SRL is the ability to consciously conduct learning strategies that are actively initiated by the students themselves rather than externally provided or instructed from educators or faculties [37]. SRL skills are not equally shared by all students at the beginning of their academic career, but they can be continuously learned and practiced. Teaching SRL may be an option to facilitate the adaptation to the challenges and hardships of the new e-learning curriculum during the COVID-19 pandemic [38].

Students can differentiate between different qualities of e-learning offerings as they value sophisticated screencasts and webinars more than simple lecture notes. In addition, they can also differentiate between areas in which e-learning can serve as an improvement and areas in which it has clear limits. From their perspective, lectures and—to some degree—seminars could be easily translated into online offerings, whereas practical skills can only be taught in person. Similar results have been found in the literature, which has reported that the format of the course—online or in-person—does not affect the acquisition of knowledge [39], whereas a difference is apparent in learning practical skills, forming identity, and adopting a professional role as a physician [40]. Practical skills, early hands-on experience and patient contact are difficult to teach or learn via remote learning. From literature and own experience, these practical aspects of medical teaching can substantially motivate students for their studies and form their perception as physicians [41]. As seen in our survey, students hold the view that the acquisition of practical skills cannot be adequately substituted by e-learning alternatives. However, various concepts have been proposed that aim to combine the advantages of both in-person and online modules for improved student satisfaction and results [42, 43]. These concepts frequently have in common that they try to build a broad theoretical background in a time-efficient manner for the practical application of the newly acquired knowledge. Therefore, e-learning could serve as a flexible adjunct in the curriculum for teaching practical skills. Examples include studying the theoretical background of certain surgical skills via e-learning [44, 45], expanding on clinical problem-solving skills [46, 47], and preparing for challenging patient interactions [48]. Especially hybrid teaching formats could play a stronger role in medical education in the future. Faculties have invested in and set up the infrastructure for online teaching and both students and educators have gained sufficient experience with its application. Hybrid formats could provide “the best from both worlds”, namely, that is the highly appreciated flexibility and the opportunity for direct contact among peers and teachers. However, providing the infrastructure for both formats could mean the less efficient use of teachings resources.

Improvements in medical education are not an end in and of themselves; indeed, they should ideally lead to increased knowledge and skills of soon-to-be physicians and—in the long run—to better patient care and clinical outcomes [49]. The benefits of e-learning with respect to better or more-efficient studying have been debated for quite some time [50–52]. A recent Cochrane Database review from 2018 found no significant improvements in clinical care when analyzing 16 randomized controlled trials and stated that e-learning is generally not more effective than traditional learning [53]. We also found equal results for the multiple-choice examination of the current semester compared with the proceeding years. However, most of our students noted subjective benefits for their learning efficacy and success and desired more e-learning courses in their curriculum in the future. Learning efficacy and independence are important not only due to the expanding medical curriculum but also with regard to students’ motivation for their later specialty training independent of their gender [54]. Evoking interest in a specialty is crucial in a time with a growing shortage of physicians and an accelerating competition for graduate students [55, 56].

### Limitations

Interpretating our results may be limited by the fact that our study was single-centered and evaluated the e-learning modules of one single specialty. The questionnaire was used for the first time and had not been previously validated. We found no particularly high interest in the specialty of obstetrics and gynecology among our students, which indicates that no positive bias for the course evaluation was present. Another limitation was the ongoing COVID-19 pandemic itself. Students have been challenged with serious issues concerning their mental health and well-being. A German survey from 2021 revealed that among 3382 students, 1294 (37%) of them reported potentially clinically relevant depressive symptoms during the COVID-19 pandemic. However, especially medical students demonstrated a higher degree of mental and physical resilience compared to students in other fields, such as in humanities or natural sciences. Therefore, it is not clear how much these social problems have potentially affected the perception of our course (57). Students could not choose between e-learning or conventional in-person courses. Due to the ongoing challenges of everyday life during the pandemic, it is not possible to rule out its impact on the benefits of remote learning. However, as e-learning reflects the continuous advancement of digital communication in everyday life, it is reasonable to assume that e-learning can be approved for use in standard curricula. Future studies under normal conditions are needed to elaborate on our findings.

## Conclusion

Our data demonstrate that the traditional teaching format of face-to-face lectures does not meet the flexibility demanded by today’s generation of medical students. E-learning, on the contrary, is practical and appreciated by our students and leads to equivalent test results compared with regular teaching methods at a German university hospital. Apart from possible setbacks in overcoming COVID-19 in the near future, various e-learning formats might be feasible tools for making medical education more student-orientated. Students highly value the teaching quality of lectures as a means of transferring knowledge; however, the face-to-face format also frequently deters them from participating. E-learning formats—such as screencasts with audio narration or online webinars—might serve as time-efficient alternatives and thus leave more time for small-group teaching and bedside learning, thereby ultimately improving future patient care. Building on the experiences gained during the COVID-19 pandemic, we want to expand our online offerings in the nearer future. Possible implications could be the regular transformation of face-to-face lectures into online or hybrid live webinars, problem-based-learning via e-learning for better preparation of practical skill modules, or new and innovative mobile app-based teaching formats.

## Data Availability

All (raw) data and material are available upon reasonable request.
